# Effects of ocean acidification on embryonic respiration and development of a temperate wrasse living along a natural CO_2_ gradient

**DOI:** 10.1093/conphys/cov073

**Published:** 2016-02-26

**Authors:** Carlo Cattano, Folco Giomi, Marco Milazzo

**Affiliations:** Department of Earth and Marine Sciences (DiSTeM) and CoNISMa, University of Palermo, Via Archirafi 28 I-90123, Palermo, Italy

**Keywords:** Early development, global change, physiological performance, *Symphodus ocellatus*, temperate fish

## Abstract

Volcanic CO_2_ seeps provide opportunities to investigate the effects of ocean acidification on organisms in the wild. To understand the influence of increasing CO_2_ concentrations on the metabolic rate (oxygen consumption) and the development of ocellated wrasse early life stages, we ran two field experiments, collecting embryos from nesting sites with different partial pressures of CO_2_ [*p*CO_2_; ambient (∼400 µatm) and high (800–1000 µatm)] and reciprocally transplanting embryos from ambient- to high-CO_2_ sites for 30 h. Ocellated wrasse offspring brooded in different CO_2_ conditions had similar responses, but after transplanting portions of nests to the high-CO_2_ site, embryos from parents that spawned in ambient conditions had higher metabolic rates. Although metabolic phenotypic plasticity may show a positive response to high CO_2_, it often comes at a cost, in this case as a smaller size at hatching. This can have adverse effects because smaller larvae often exhibit a lower survival in the wild. However, the adverse effects of increased CO_2_ on metabolism and development did not occur when embryos from the high-CO_2_ nesting site were exposed to ambient conditions, suggesting that offspring from the high-CO_2_ nesting site could be resilient to a wider range of *p*CO_2_ values than those belonging to the site with present-day *p*CO_2_ levels. Our study identifies a crucial need to increase the number of studies dealing with these processes under global change trajectories and to expand these to naturally high-CO_2_ environments, in order to assess further the adaptive plasticity mechanism that encompasses non-genetic inheritance (epigenetics) through parental exposure and other downstream consequences, such as survival of larvae.

## Teaser

We assessed rising CO2 effects on metabolism and development of a nesting wrasse by reciprocal transplant experiments in the field. Offspring brooded under different CO2 conditions exhibited similar responses. However, embryos from High-CO2 site were resilient to a wider range of pCO2 levels than those belonging to current-day conditions.

## Introduction

There is widespread concern that the increase of dissolved CO_2_ concentrations in the oceans and the consequent alteration of the water chemistry (ocean acidification, OA) may severely affect a wide range of marine organisms (Kroeker *et al.*, 2010, 2013; Wittmann and Pörtner, 2013). Modelling projections based on CO_2_ emission scenarios proposed by the Intergovernmental Panel on Climate Change (IPCC) suggest that if global emissions will not be reduced, ocean CO_2_ partial pressure (*p*CO_2_) will continue to increase up to ∼1000 µatm by the end of this century, and the average surface ocean pH will drop by up to 0.4 units compared with present-day levels (Pörtner *et al.*, 2014).

In the last decade, the number of studies dealing with the effects of OA on the marine biota has constantly increased, suggesting OA as one of the most serious threats for marine organisms and ecosystem functions (Kroeker *et al.*, 2010). However, a wide variability of responses to OA have been observed among different taxonomic groups and ontogenetic stages, with calcifying organisms considered the most threatened taxa (Kroeker *et al*., 2013).

Being able to regulate their internal acid–base balance actively, fish have been long considered to be less sensitive to changes in the water carbonate chemistry (e.g. Melzner *et al.*, 2009b). However, sublethal effects were displayed by fish exposed to high *p*CO_2_ levels, especially during early life stages. It has been suggested that fish exposed to high CO_2_ concentrations have to adjust the internal HCO_3_^−^ to maintain homeostasis, with this having consequences on ionic regulation processes (e.g. Brauner and Baker, 2009; Esbaugh *et al*., 2012; Heuer and Grosell, 2014). This regulation in response to high CO_2_ concentrations is predicted to be energetically costly (Pörtner *et al.*, 2004; Munday *et al*., 2009a) and may lead to altered survival rates (Baumann *et al*., 2012; Chambers *et al*., 2014), reproduction (Miller *et al.*, 2013), development (Frommel *et al*., 2012; Miller *et al*., 2012; Forsgren *et al*., 2013) and calcification (Checkley *et al*., 2009; Munday *et al*., 2011; Pimentel *et al*., 2014a) in early life stages of some fish species. In addition, an increasing number of studies have documented effects of altered *p*CO_2_ levels on the behaviour and neurosensory functions of different fish species, such as impaired learning, loss of behavioural lateralization and altered auditory and olfactory abilities (Munday *et al.*, 2009b, 2010, 2014; Simpson *et al*., 2011; Domenici *et al*., 2012; Nilsson *et al*., 2012; Jutfelt *et al*., 2013; Chivers *et al*., 2014; Heuer and Grosell, 2014). The molecular mechanism underpinning such responses has been elucidated by Nilsson *et al*. (2012), who demonstrated how the function of the GABA_A_ receptor, an inhibitory neurotransmitter of the vertebrate brain, is affected by higher CO_2_ concentrations.

Physiological studies revealed that adult fish display an apparently high tolerance even at very high *p*CO_2_ levels that will not be reached in any worst-case OA scenario (Ishimatsu *et al*., 2008), whereas early life stages seem to be threatened at *p*CO_2_ levels expected by the end of this century (i.e. ∼1000 µatm) or beyond (Baumann *et al*., 2012; Frommel *et al*., 2012; Forsgren *et al*., 2013; Chambers *et al*., 2014; Pimentel *et al.*, 2014a). The higher sensitivity of early life stages has been linked to their high volume-to-surface ratio affecting the diffusive processes and to an acid–base balance system not yet fully developed, with ionic exchanges occurring across the skin and the yolk (Ishimatsu *et al*., 2008). It has also been suggested that the energetic cost of maintenance of acid–base balance in high-CO_2_ conditions would increase the metabolic rate, with potential consequences for development, performance and survival of subsequent ontogenetic stages (Sokolova *et al*., 2012). To date, studies on fish metabolic responses to altered levels of *p*CO_2_ were mainly carried out on larval and post-larval stages and often showed mixed responses (Melzner *et al*., 2009a; Munday *et al*., 2009a, 2014; Miller *et al*., 2012; Couturier *et al*., 2013; Enzor *et al*., 2013; Rummer *et al*., 2013; Pimentel *et al*., 2014b; Pope *et al*., 2014). Only a few studies addressed the combined effects of CO_2_ and temperature on potential alterations of fish embryonic physiology, such as the metabolic response of the little skate, *Leucoraja erinacea* (Di Santo, 2015), and the Antarctic dragonfish, *Gymnodraco acuticeps* (Flynn *et al*., 2015).

More importantly, most of the knowledge about the effects of OA is derived from studies performed in laboratory conditions, whereas the responses of organisms in their natural habitats have seldom been verified, although this could better reflect their adaptation to natural variability in pH and *p*CO_2_ (Munday *et al*., 2014) and to long-term selective pressure. Volcanic CO_2_ seeps represent a suitable situation in which to disclose these responses and have been used recently to address fish behavioural responses and potential transgenerational effects on populations chronically exposed to high-CO_2_ conditions (Munday *et al*., 2014).

Here, we performed two experiments in a natural CO_2_ seep off Vulcano Island (Italy) to assess the effects of different *p*CO_2_ levels on the early development of the temperate ocellated wrasse, *Symphodus ocellatus* (Forsskål, 1775). This species was chosen because it was previously observed spawning along the CO_2_ gradient and its eggs are laid on benthic nests. In the first experiment, we used a factorial design to assess the oxygen consumption of three embryonic stages (i.e. initial, middle and late) reared in nesting sites in present-day ambient conditions (ambient, A) and end-of-century high-CO_2_ conditions (high CO_2_, H). Embryos were also transplanted from the high-CO_2_ nesting site to the ambient site and vice versa (HA and AH, respectively) or replaced into the original nesting site (HH and AA) to control the translocation effect, and then exposed for 30 h before testing. This enabled us to assess the effects of elevated CO_2_ on egg size and oxygen consumption of the *S. ocellatus* embryos and their responses when developed in different CO_2_ conditions from the original nesting site. In the second experiment, using the same factorial design, we assessed how different *p*CO_2_ levels may affect the yolk consumption and length of newly hatched larvae.

## Materials and methods

### Study species

*Symphodus ocellatus* is a Mediterranean wrasse widespread in shallow rocky areas. This species is a benthic spawner, and the females lay eggs in nests built by territorial males (nesting males or nest-builders; Taborsky *et al*., 1987). The breeding season usually occurs from April to July, probably according to seawater temperature (Lejeune, 1985). During the reproductive period, the nesting males become strictly territorial and brightly coloured and build nests with fragments of algae, where they attract females for mating. The sexual activity of this species is recurrent, and each nesting male completes several nesting cycles over the breeding season (Taborsky *et al*., 1987). Females visit different nests and lay tens of small eggs (approximately 10–40 eggs) for each spawning event (Lejeune, 1985). The eggs are immediately fertilized, and nesting males provide care for the embryos until hatching, moving the pectoral fins above the nest surface. Development of embryos takes up to 80 h at 21°C (Lejeune, 1985), whereas the subsequent pelagic larval phase lasts from 9 to 13 days (with average planktonic larval duration of 10 days as documented by settlement marks in otoliths; Raventos and McPherson, 2001).

### Study site

The experiments were carried out in two different breeding seasons (early and late May 2012 and June 2013) in the Baia di Levante of Vulcano Island (Aeolian Archipelago, Northeastern Sicily, Italy). In this area, a submersed CO_2_ seep system generates a CO_2_/pH gradient that runs parallel to the coast (Boatta *et al*., 2013). The main submersed seep is located along southern and western shores of Baia di Levante (38°25′03.07″N, 14°57′35.90″E) at 1 m depth, and the gas composition is mainly CO_2_ (>99%). Other gases potentially toxic for cell respiration (e.g. H_2_S) are found at the main seeping site but do not extend to the nesting sites we considered in the present study, which are located at a distance of >400 m from the main degassing area (Boatta *et al*., 2013). Oxygen concentrations reach ambient conditions at a few tens of metres from the main seeps (Boatta *et al*., 2013). Two nesting sites with different *p*CO_2_ and pH levels were identified for the experiments: ambient CO_2_ (A) in present-day conditions (∼400 µatm, *p*CO_2_) and high CO_2_ (H), mimicking projected end-of-century CO_2_ conditions (∼1000 µatm *p*CO_2_ for the first experiment and ∼800 µatm *p*CO_2_ for the second experiment; see online supplementary material Table S1 and Fig. S1; RCP8.5 scenario; IPCC, 2013).

### Experiment 1: effects of different CO_2_ concentrations on oxygen consumption of embryos

In each nesting site exposed to different CO_2_ conditions, we identified 10 nests where the dominant nesting male was in the spawning phase, and we collected portions of the nests for subsequent 30 h exposure. Specifically, fragments of nests with eggs were moved from the high-CO_2_ nesting site to the ambient-CO_2_ site and vice versa (treatments HA and AH, respectively; five nests for each condition) or replaced into the original nesting site (treatments HH and AA; five nests for each condition) to control the translocation effect. Translocations were performed by placing the portions of each nest in 100 ml plastic containers with four sides opened and covered with a mesh (0.1 mm) to ensure water flow-through and oxygenation of the embryos. The containers were fixed to the bottom for 30 h at the same depth as the nests of provenience (3–4 m). After the 30 h exposure, eggs from the A and H nesting sites were also collected from eight additional nests.

Embryos belonging from the six different treatments (A, H, AH, AA, HA and HH) were classified on the basis of their development stages, as follows: the first stage (initial) included embryos at the end of epiboly phase (∼6–8 h after fertilization); the second stage (middle) included embryos from the end of the epiboly phase to the formation of the last somite (∼10–30 h after fertilization); and the last stage (late) started with the exhibition of body and eye pigmentation and progressed until the pre-hatching phase (∼60–80 h after fertilization). As expected, only embryos belonging to the middle and late stage of development were found in the AH, AA, HA and HH treatments after the 30 h exposure.

The oxygen consumption rate (OCR) was measured for groups of 10 embryos from each treatment and developmental stage. To measure the OCR, we used the electrode microrespiration System, MRS-8 (Unisense, Aarhus, Denmark), provided with eight microrespiration glass chambers containing ∼0.5 ml (Bartolini *et al*., 2013; Simoni *et al*., 2013). To ensure the constant homogeneity of the water samples, each chamber was stirred with a glass-embedded micromagnet (separated from the eggs through a fine mesh of stainless steel) and an individual stirring device. Respirometric chambers were filled with filtered (0.2 µm) seawater to reduce the confounding effect of microorganismal respiration. The temperature of the system was maintained at the same temperature recorded in the field during the first experiment (see supplementary Fig. S2). When the system stabilized at the experimental temperature, 10 eggs were introduced to the glass chambers and oxygen concentration was measured. An end-point measurement was performed after 2 min, and the OCR was extrapolated from the difference between the two measurements (i.e. oxygen depletion/time) and was reported as the absolute value. The approximate single embryo OCR was estimated by dividing the oxygen consumption by the number of embryos in the respirometric chamber. After measuring the oxygen consumption, we placed the eggs on a Petri dish with a 1 mm grid with 0.05 mm scale bars and we photographed them with a Leica camera attached to a stereo microscope (Leica MZ APO). Egg surface area was obtained from digital photographs using ImageJ software by tracing around each egg, and the area was estimated to the nearest 0.001 mm^2^. As the difference in size of eggs could influence the amount and the capacity of gas exchange of the embryos tested for the respirometry, we standardized the oxygen consumption to the average egg surface calculated for each replicate (10 embryos) and thus reported oxygen consumption in micromoles per hour per square millimetre.

### Experiment 2: effects of different CO_2_ concentrations on yolk area and length at hatching

In June 2013, a second experiment was performed to evaluate whether exposure to different CO_2_ conditions may affect the yolk consumption (i.e. yolk area at hatching) and the length of larvae at hatching. We used the same experimental design and the same methods as in the first experiment. Portions of nests were collected from A and H nesting sites (five nests per site), and portions of five nests each containing some hundreds (appoximately 500–700) of embryos were exposed to each of the translocation treatments (AH, HA, HH and AA; 20 portions of nests in total). In this case, we chose to focus collections on nests in the last stage of the nesting cycle (when the nesting male provides parental care by fanning) to be sure to obtain enough hatchlings for subsequent measurements. After the 30 h exposure, the nest portions were collected from each CO_2_ treatment and transferred to a laboratory facility close to the field site, where they were placed in six different rearing aquaria (10 l each) and maintained in aerated seawater from the site of origin. The temperature was monitored using a 556 MPS YSI (Yellow Springs, OH, USA) multiprobe.

Hatching is synchronous in this species and occurs at dusk, probably to avoid predation (Lejeune, 1985). Hatchlings were collected from each aquarium within 2 h, photographed under a binocular microscope (Leica, MZ-APO) and then released in the nesting sites of origin. Yolk area and total length of the larvae were estimated to the nearest 0.001 mm^2^ and 0.01 mm, respectively, using the digital photographs and the image analysis software ImageJ.

### Seawater carbonate chemistry

For the first experiment, salinity and pH expressed as total scale (pH_T_) were measured *in situ* for 3 days consecutively in early and late May 2012, using a 556 MPS YSI multiprobe positioned at 2 m depth and previously calibrated using Tris–HCl and 2-aminopyridine–HCl buffer solutions (Dickson *et al*., 2007). Hobo Onset loggers were also deployed in the two nesting sites to monitor seawater temperatures (in degrees Celsius) continually at 1 h intervals for the whole duration of the first experiment (supplementary Fig. S2). Dissolved oxygen (DO, in milligrams per litre) was measured only in late May (*n* = 6 for each nesting site). At both sites, 100 ml water samples (*n *= 3) were also collected, passed through a Whatman GF/F, poisoned with 0.05 ml of 50% HgCl_2_ (Merck, Analar) and stored in the dark at 4°C for subsequent analyses of total alkalinity (TA). Three replicate 20 ml sub-samples were analysed at 25°C using a titration system composed of a pH meter with a Methrom pH electrode and a 1 ml automatic burette (Methrom). The pH was measured at 0.02 ml increments of 0.1 N HCl. Total alkalinity (in micromoles per kilogram) was calculated from the Gran function applied to pH from 4.2 to 3.0, as milliequivalents per litre from the slope of the curve for pH vs. HCl volume. Results were corrected against TA standards provided by A.G. Dickson (batch 99 and 102). For the first experiments, parameters of the carbonate system [*p*CO_2_, CO_3_^2^ and HCO_3_^−^] were calculated from pH_T_, average TA, temperature and salinity using the free-access CO_2_SYS package (Pierrot and Wallace, 2006). Means of pH_T_ were calculated from hydrogen ion concentrations of each measurement and then reconverted back to pH.

For the second experiment, *p*CO_2_ (in microatmospheres) was continuously measured by deploying a HidroC^tm^CO_2_ II sensor for dissolved CO_2_ (Contros System & Solutions GmbH, Germany) at 2 m depth at each nesting site for 20 h, whilst salinity and seawater temperature (in degrees Celsius) were monitored on several visits using a 556 MPS YSI.

The seawater carbonate chemistry of the first experiment and the *p*CO_2_ measured during the second experiment are presented as online supplementary material (supplementary Tables S1 and S2 and Fig. S1).

### Experimental design and data analyses

Potential differences in egg size, OCR, yolk area and length at hatching were tested by permutational univariate analysis of variance (PERMANOVA; Anderson *et al*., 2008) using PRIMER v. 6.1 (PRIMER-E, Ivybridge, UK). Differences in egg size were tested using ‘nesting site’ as a fixed factor with two levels (H and A) to assess potential effects of different *p*CO_2_ levels on eggs laid at different nesting sites, or ‘period’ as a fixed factor with two levels (early May and late May) to assess potential differences across the breeding season.

Differences in the overall oxygen consumption of embryos among the three developmental stages were then determined by a two-factor design, with ‘embryonic stage’ as a fixed factor with three levels (initial, middle and late) and ‘nesting site’ as a fixed orthogonal factor with two levels (H and A). Within each embryonic stage, differences in OCR were tested using ‘CO_2_ treatment’ as a fixed factor with two levels (H and A) for the initial developmental stage, five levels for the middle developmental stage (H, A, AH, HA and AA; as we were not able to obtain enough middle-stage embryos for the HH treatments) and with six levels (H, A, AH, HA, HH and AA) for the late developmental stage. Differences in length at hatching were determined using ‘treatment’ as a fixed factor with six levels (A, H, AH, HA, AA and HH). Data for each experimental trials were not transformed, and a triangular matrix based on Euclidean distance was calculated for each data set. One-way and two-way PERMANOVAs were run using 9999 unrestricted permutations of the raw data. Pairwise *t*-test comparisons of significant terms in PERMANOVAs were used to assess differences between levels. A linear regression analysis was also run to assess the relationships between the three development stages of *S. ocellatus* embryos from ambient- and high-CO_2_ nesting sites and their oxygen consumption (in micromoles per hour per square millimetre).

## Results

**Figure 1:**
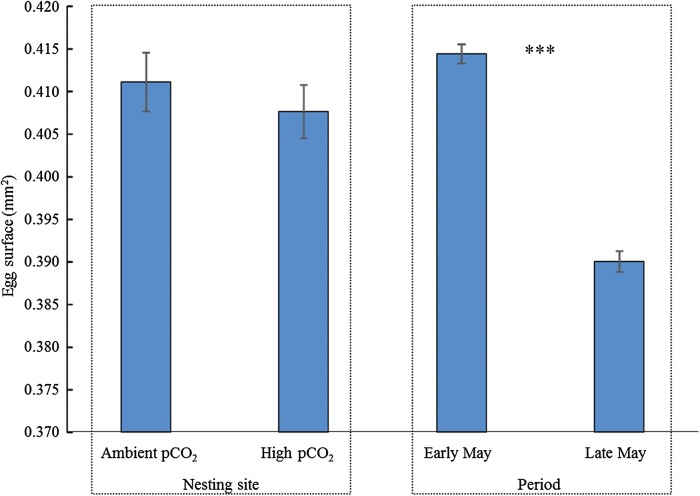
Mean (±SEM) surface area of newly fertilized eggs collected in the first experiment from nesting sites with ambient (A) and high (H) CO_2_ (left panel) and overall mean (±SEM) surface area of eggs regardless of the stage of development in the two periods (early and late May; right panel). In this latter case, eggs belonged to both CO_2_ nesting sites. ***P = 0.001. *n* = 88 (A), *n* = 87 (H), *n* = 769 (early May) and *n* = 456 (late May).

In the first experiment, the average size of newly fertilized eggs did not differ between A and H conditions (means ± SEM: A, 0.411 ± 0.003 mm^2^; H, 0.408 ± 0.003 mm^2^; PERMANOVA, pseudo-*F*_1,174 _= 0.55209, *P* = 0.4476; Fig. 1). The eggs collected in late May were significantly smaller than eggs collected in early May in both ambient- and high-CO_2_ sites (means ± SEM: early May, 0.414 ± 0.001 mm^2^; late May, 0.390 ± 0.001 mm^2^; PERMANOVA, pseudo-*F*_1,1224 _= 202.99, *P* < 0.001; Fig. 1).

**Figure 2:**
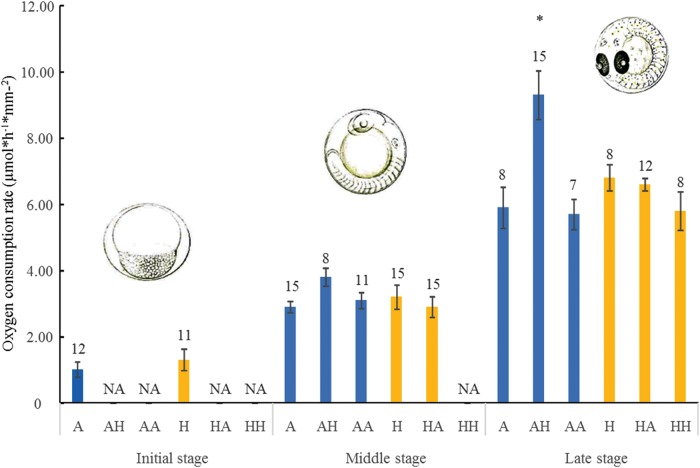
Oxygen consumption rate of embryos standardized to egg surface area (in micromoles per hour per square millimetre). Embryos were reared in nesting sites at ambient conditions (A) and end-of-century high-CO_2_ conditions (H), transplanted from the high-CO_2_ to the ambient nesting site and vice versa (HA and AH, respectively), or replaced into the original nesting site (HH and AA) to control the translocation effect. Graph shows the average (±SEM) O_2_ consumption in each treatment and developmental stage (results for HH treatments in the middle stage were not available). Numbers above bars indicate the number of replicates. Asterisk (*) indicates the presence of significant differences (at P < 0.05) (see also pairwise tests in supplementary Table S4). NA, not available.

The OCRs of embryos collected from high- and ambient-CO_2_ nesting sites increased linearly as development progressed, from the initial to the late stage (ambient CO_2_, *r*^2^ = 0.7559, *P* < 0.001; high CO_2_, *r*^2 ^= 0.7234, *P* < 0.001; supplementary Fig. S3). At each developmental stage, we found no differences in the OCR between embryos collected in A and H conditions (Fig. 2; PERMANOVA: pseudo-*F*_1,68 _= 2.959, *P* = 0.0903; supplementary Table S3). Also, these treatments were not different from the translocation controls, HH and AA, in the middle and late developmental stages and from embryos belonging to high-CO_2_ and moved to ambient-CO_2_ nesting sites (HA) for 30 h exposure (Fig. 2; supplementary Table S4). Embryos collected in A and exposed to H conditions (i.e. AH treatment) showed an increasing trend of oxygen consumption in the middle stage and a significantly higher OCR for embryos in the late developmental stage (Fig. 2 and supplementary Table S4).

**Figure 3:**
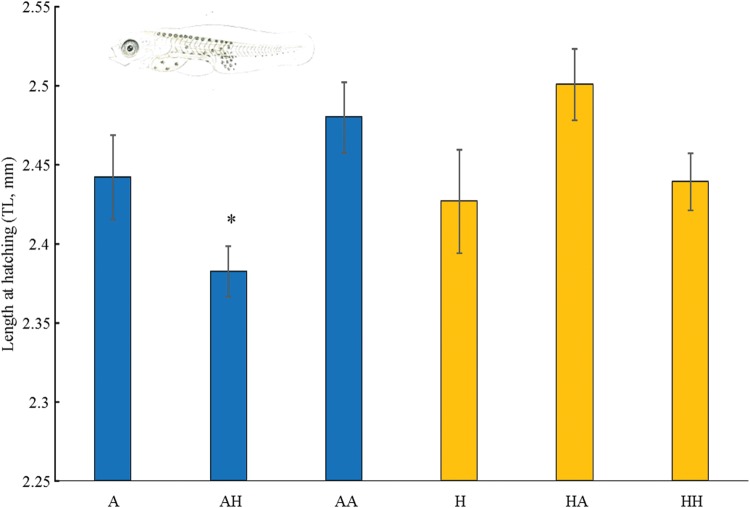
Mean (±SEM) total length (in millmetres) of larvae hatched from embryos exposed to the six treatments. Asterisk (*) indicates the presence of significant differences (at *P* < 0.05). *n* = 48 (A), *n* = 72 (AH), *n* = 84 (AA), *n* = 62 (H), *n* = 66 (HA) and *n* = 82 (HH).

In the second experiment, the length of newly hatched larvae did not differ between A and H nesting sites (PERMANOVA: pseudo-*F*_5,413 _= 3.3936, *P* = 0.0052; supplementary Table S5), where the total length of the larvae were on average 2.44 ± 0.03 and 2.42 ± 0.03 mm, respectively (means ± SEM; Fig. 3). Larvae hatched from AA and HH treatments showed no significant differences from A and H treatments, as total length was on average 2.48 ± 0.02 mm in the AA treatment and 2.43 ± 0.01 mm in the HH treatment (Fig. 3 and supplementary Table S5). Larvae hatched from the AH treatment were significantly smaller (2.38 ± 0.01 mm) than those in all the other treatments (supplementary Table S5).

The yolk area of the newly hatched larvae showed a high variability among the treatments (PERMANOVA: pseudo-*F*_5,413 _= 34.485, *P* = 0.001; supplementary Fig. S4). The yolk area in all the treatments differed significantly from each other except those belonging to the HA and HH treatments (supplementary Table S6). On average, the largest yolk area was recorded in the AH treatment (0.109 ± 0.003 mm^2^) and the smallest in the H treatment (0.065 ± 0.001 mm^2^; supplementary Fig. S4).

## Discussion

We show that embryos of the ocellated wrasse (*S. ocellatus*) living at a temperate CO_2_ seep are tolerant to CO_2_ concentrations expected by the end of this century. Specifically, the oxygen consumption of embryos and their size at hatching were similar between ambient- and high-CO_2_ nesting sites. However, we found an altered respiration rate in embryos collected from an ambient-CO_2_ and moved to a high-CO_2_ nesting site for a 30 h exposure, implying that embryos from ambient-CO_2_ conditions could be less able to cope with higher *p*CO_2_ levels. Likewise, hatchlings showed an impaired development in these conditions. On the contrary, embryos belonging to the high-CO_2_ nesting site showed no such response when translocated to the ambient-CO_2_ treatment, therefore suggesting that offspring from high-CO_2_ nesting sites could be resilient to a wider range of *p*CO_2_ values than those belonging to the site with present-day *p*CO_2_ levels.

Embryos reared in ambient-CO_2_ conditions experienced a lower variability in pH and *p*CO_2_ than embryos reared in high-CO_2_ conditions off the Vulcano Island seep in both experiments. It has been suggested that through maternal provisioning females may adjust egg characteristics (such as optimal egg size; Einum *et al*., 2002) to environmental conditions (Chambers, 1997). However, we found that the size of *S. ocellatus* eggs laid in the two nesting sites did not differ, whereas we documented a decrease in egg size during the breeding season. Egg surface is often used as indicator of egg quality in fish and represents a fundamental life-history trait for marine fish that influences the size of embryos and their physiology, growth rate and survival at hatching, with cascading effects on species performances and fitness (Chambers, 1997). Our findings suggest that *S. ocellatus* could modulate their investment in ways other than egg dimension. Likewise, no differences were found in the size of eggs laid from parents of the cinnamon anemonefish (*Amphiprion melanopus*) reared in aquaria at *p*CO_2_ levels comparable with those considered in the present study (Miller *et al*., 2013). The observed differences in egg size throughout the breeding season have already been documented for several temperate spring-spawning species (e.g. Chambers, 1997; Mazzoldi *et al*., 2002), including the co-generic wrasse *Symphodus roissali* (Raventos and Planes, 2008), and have been related to the body condition and the size of the females (Marshall *et al.*, 2010).

As fish primarily use metabolic adjustments to cope with acid–base disturbances (Heuer and Grosell, 2014), metabolic responses measured as OCR may give a clear indication of the physiological performance and potential resilience of organisms exposed to different *p*CO_2_ levels (Sokolova *et al*., 2012). As suggested by some authors, when the *p*CO_2_ increases and pH drops, respiration rate increases in order to exhale CO_2_ and restore homeostasis, affecting the whole energy budget of the organisms (Ishimatsu *et al*., 2008; Pörtner, 2008; Munday *et al*., 2009a). Other authors reported metabolic depression in fish exposed to high *p*CO_2_ levels (Rummer *et al.*, 2013; Pimentel *et al.*, 2014b), probably because of a mechanism of protection of the body fluids from excessive acidification (Rummer *et al.*, 2013). As expected, we recorded an increased oxygen consumption throughout the embryonic development. Given that the oxygen consumption of embryos in initial, middle and late developmental stages did not show any difference between ambient- and high-CO_2_ sites, we suggest that offspring of *S. ocellatus* from parents living in altered CO_2_ conditions could be resilient to high *p*CO_2_. This result could indicate that parental effects (i.e. when parents and offspring are exposed to the same CO_2_ levels) can compensate for the effects of high CO_2_ on embryonic metabolic performance by conditioning the offspring to specific CO_2_ levels through epigenetic transgenerational plasticity, allowing offspring to develop efficient physiological pathways for the high-CO_2_ environment (e.g. Miller *et al*., 2012). As *S. ocellatus* is highly territorial during the breeding season and embryos develop in benthic nests (Taborsky *et al*., 1987), both parents and offspring were exposed to the same environmental conditions off Vulcano Island CO_2_ gradient. However, the 30 h exposure of embryos collected at ambient-CO_2_ site and moved to higher levels of *p*CO_2_ (high-CO_2_ nesting site) led to an increase of OCR, which was more evident during the late developmental stage (with embryos showing an average 33.8% increase in OCR relative to embryos exposed to other treatments). As oxygen consumption may be considered a proxy of energetic demand for basal maintenance and development, our results could suggest that embryos from parents living in ambient-CO_2_ conditions increase their oxygen requirement when they are exposed to high-CO_2_ conditions in order to support the increased acid–base regulatory activity (Heuer and Grosell, 2014). On the contrary, there is no sign of metabolic impairment in embryos moved from high-CO_2_ to ambient-CO_2_ nesting sites, indicating that this translocation does not represent a stressful event for developing embryos. Laboratory experiments revealed that Juveniles of the tropical anemonefish *Amphyprion melanopus* exposed to altered *p*CO_2_ levels display increased routine metabolic rate (Miller *et al*., 2012). However, this adverse effect of increased CO_2_ on metabolic rate did not occur when juveniles were reared in the same CO_2_ conditions as their parents, suggesting that the conditions experienced by adults may lead to improved capacity to cope with CO_2_ stress (Miller *et al*., 2012).

Similar to what has been observed for the metabolic response, the length of larvae hatched from nests at ambient and high CO_2_ did not differ between nesting sites, whereas hatchlings from embryos moved from ambient- to high-CO_2_ nesting sites were significantly smaller than those hatched in all other treatments. In the absence of an efficient regulatory system, exposure to high levels of CO_2_ might lead to an increase of internal *p*CO_2_ levels and acidification of internal fluid compartments (Heuer and Grosell, 2014). This might cause direct acid–base imbalances in the organism, which can lead to larval tissue damage (Frommel *et al*., 2012), and reallocation of energy resources away from growth or development (Baumann *et al*., 2012). Some studies assessing aspects of embryonic and larval development have shown increased or no differences in the length at hatching of larvae after exposure to high *p*CO_2_ levels (Munday *et al*., 2009c; Franke and Clemmesen, 2011; Frommel *et al*., 2013; Hurst *et al*., 2013; Miller *et al*., 2013; Chambers *et al*., 2014). This implies that the observed increment in the metabolic activity of embryos from the AH treatment could be a short-term response of transplanted embryos to support the increased energy demand for acid–base regulation in increased CO_2_ levels (Pörtner, 2012; Sokolova *et al*., 2012), potentially leading to a decreased growth rate (reduced size at hatching). A recent experiment showed that larvae of *Seriola lalandi* hatched from eggs exposed to moderate and high *p*CO_2_ levels (880 and 1700 µatm, respectively) were significantly smaller than larvae hatched from the control treatment, suggesting an additional energetic cost to cope with altered *p*CO_2_ levels (Munday *et al*., 2015). Smaller larvae may exhibit a lower performance and survival in the wild, because it has been documented that large offspring are advantaged from enhanced swimming ability and more efficient predator avoidance (Miller *et al*., 1988). However, our second experiment failed to find a consistent response when considering the yolk size at hatching. Yolk areas of larvae hatching from ambient-CO_2_ and moved to high-CO_2_ conditions were bigger than yolks in the other treatments. Further research is need to highlight potential trade-off mechanisms involving yolk consumption.

In conclusion, our study reveals that *S. ocellatus* offspring brooded in different CO_2_ conditions exhibited similar responses, but after the 30 h exposure to higher CO_2_ levels the embryos and larvae from parents that spawned in the ambient-CO_2_ nesting site showed metabolic and size differences, unlike those from parents exposed to high-*p*CO_2_ conditions. Indeed, it is undoubted that the role of acclimatization and adaptation processes will have significant consequences for our understanding of how fish will respond to a future high-CO_2_ ocean (Sunday *et al*., 2014). In this context, our study identifies a crucial need to increase the number of studies dealing with these processes under climate change trajectories and to expand these to naturally high-CO_2_ environments to assess further the adaptive plasticity mechanism that encompasses non-genetic inheritance (epigenetics) through parental exposure.

## Supplementary material

Supplementary material is available at *Conservation Physiology* online.

## Funding

This work was supported by FFR-A (2012) from the University of Palermo to M.M. and C.C., and contributes to the COST action ‘Conservation Physiology of Marine Fishes’ (FA1004).

## Supplementary Material

Supplementary Data
