# Supplementary material for: Effects of ocean acidification on embryonic respiration and development of a temperate wrasse living along a natural CO2 gradient
Source: Conserv Physiol. 2016 Feb 26;4(1):cov073. doi: 10.1093/conphys/cov073 (PMC4771110; doi:10.1093/conphys/cov073)
Supplement: Supplementary Data [file cov073supp.zip › cov073supp.pdf]

Supplementary data

Effects of ocean acidification on embryonic respiration and development of a temperate wrasse living along a natural CO_2 gradient

Carlo Cattano*, Folco Giomi, Marco Milazzo

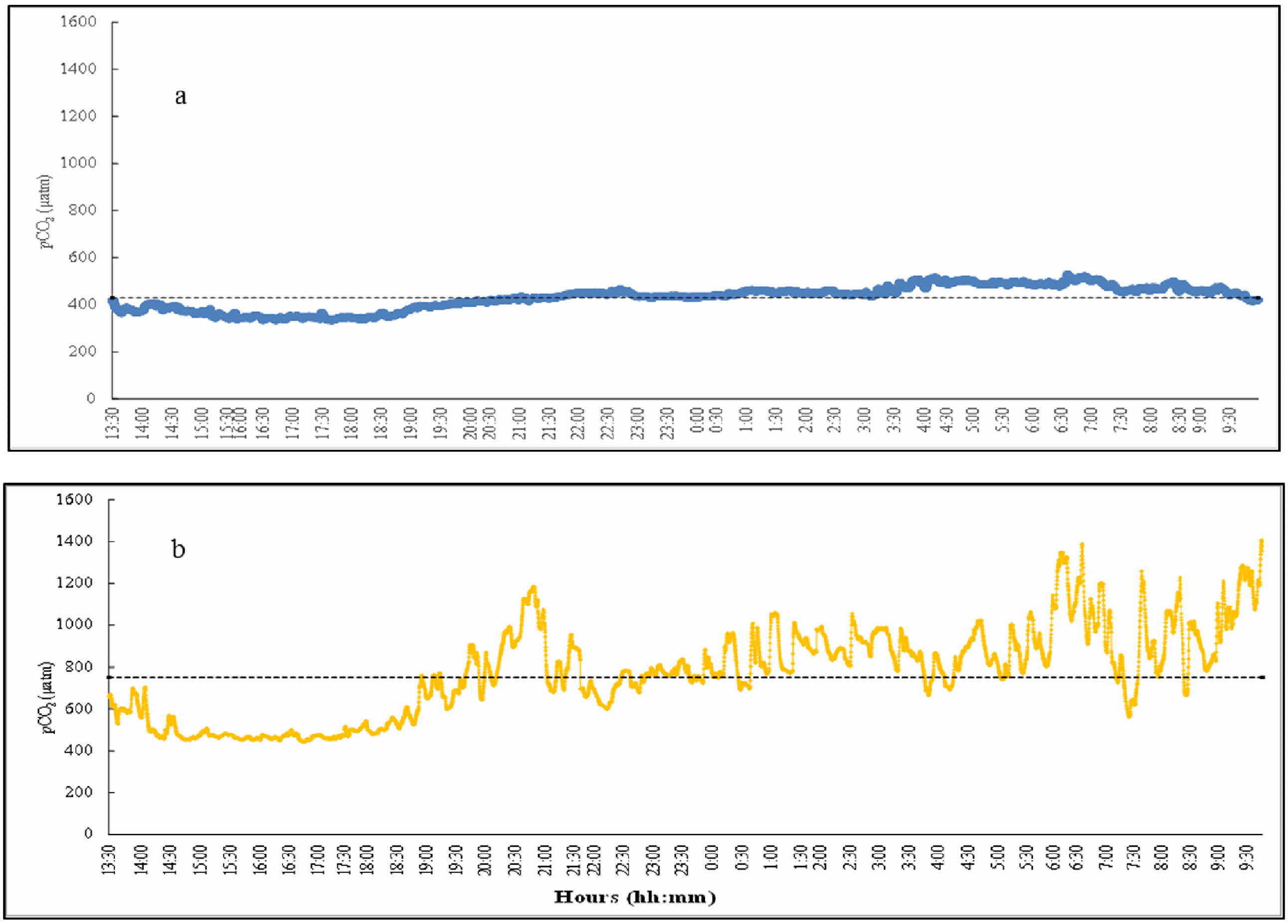

Figure S1: pCO$_2$ daily fluctuation recorded in Ambient CO$_2$ (a) and High CO$_2$ (b) nesting sites during the second experiment aimed at assessing hatchling size and yolk consumption. Dotted lines are the average values recorded during the 20-hour measure.

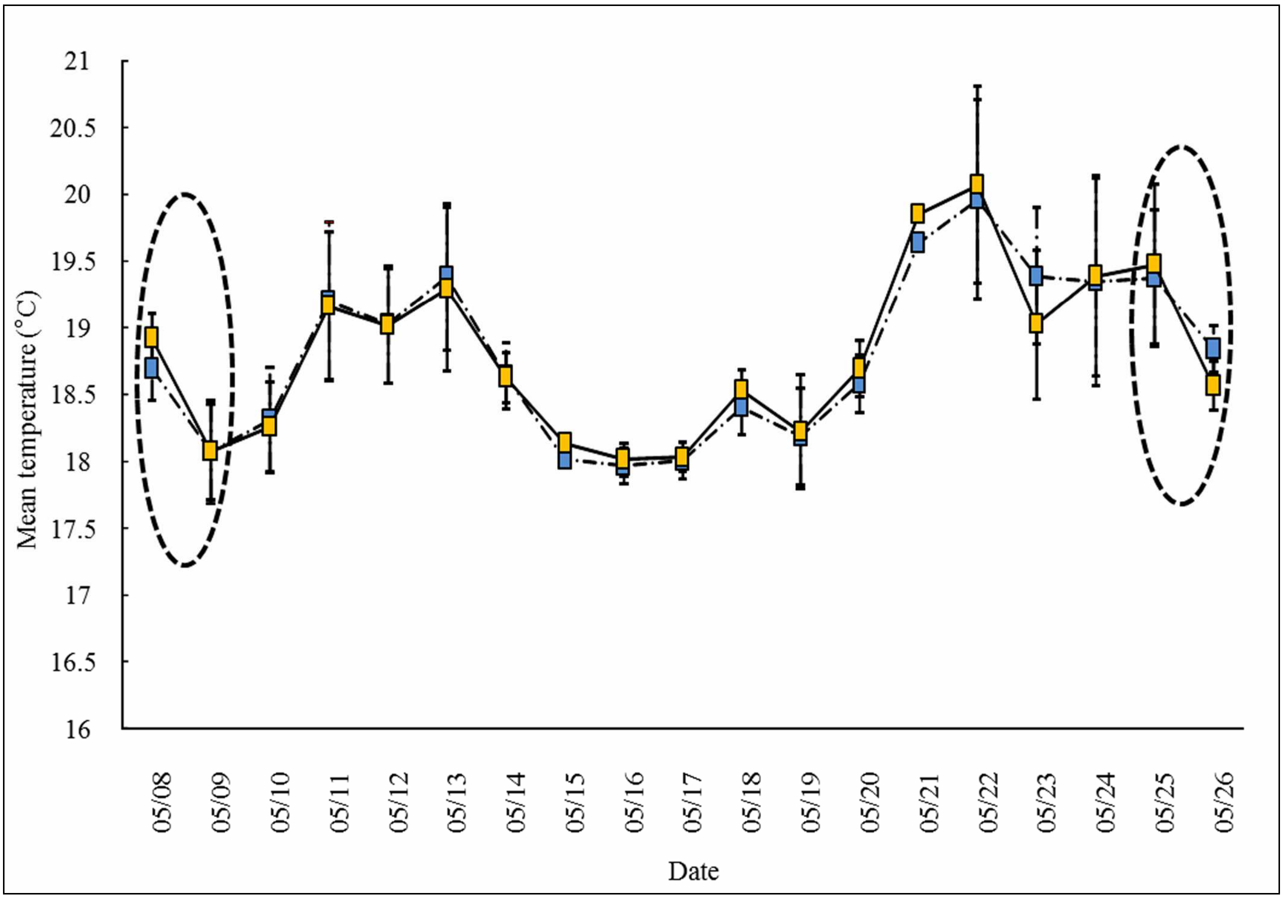

Figure S2: Mean (± SD) seawater temperature (°C) recorded in Ambient CO_2 (blue) and High CO_2 (orange) nesting sites during the first experiment (the encircled points indicates the two sampling periods).

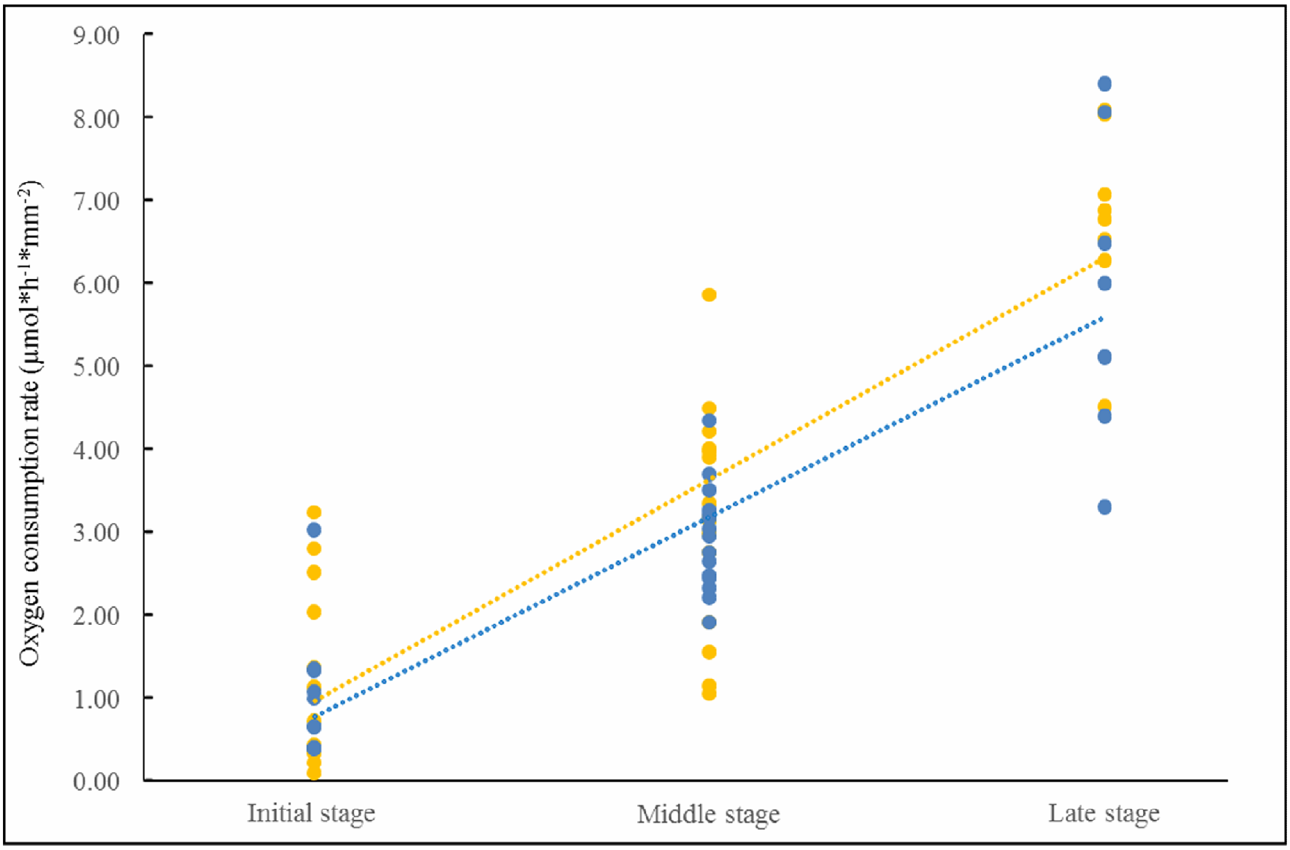

Figure S3: Oxygen consumption (µmol · h^{-1} · mm^{-2}) of the embryos at the three development stages collected from Ambient (blue) and High CO_2 (orange) nesting sites. Linear regressions (dotted lines) are reported for each CO_2 condition (Ambient CO_2: r^2 = 0.7559, p<0.001; High CO_2: r^2= 0.7234, p<0.001).

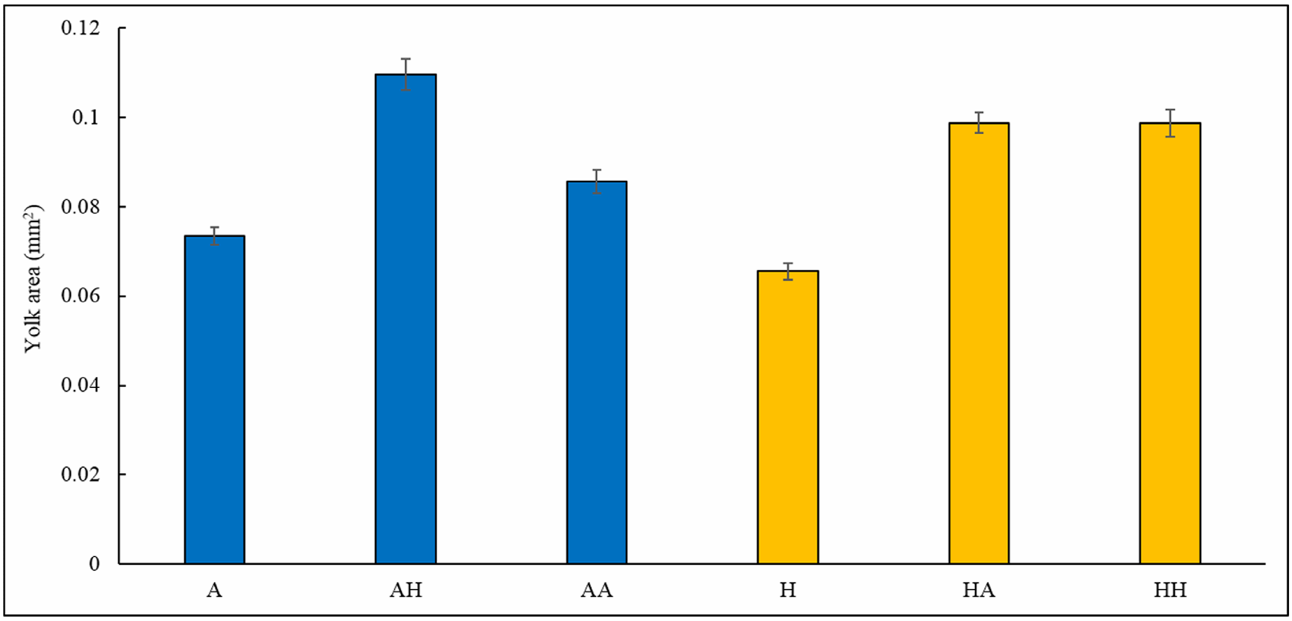

Figure S4: Mean (±SEM) yolk area (mm^2) of newly hatched larvae in the six treatments: A (n= 48), AH (n= 72), AA (n= 84), H (n= 62), HA (n= 66), and HH (n= 82). See supplementary Table S6 for full statistical differences.

Table S1: ***Seawater chemistry at the two nesting sites during the two study periods of the first experiment off Vulcano Island CO_2 seeps.***

		Nesting Sites			
		**High CO_2**		**Ambient CO_2**	
		**Early May**	**Late May**	**Early May**	**Late May**
Salinity	Mean (± SD)	38.2 (0.1)	38.2 (0.1)	38.1 (0.1)	38.1 (0.1)
Dissolved oxygen (mg/l)	Mean (± SD)	-	8.2 (0.2)	-	8.5 (0.2)
Temperature (°C)	Mean (± SD)	18.4 (0.3)	19.1 (0.5)	18.4 (0.4)	19.2 (0.3)
	Range	17.5-19.1	18.6-19.4	18.0-18.7	18.8-19.3
	Median	18.4	19.3	18.3	19.4
pH_T	Mean (± SD)	7.72 (0.19)	7.69 (0.16)	8.08 (0.02)	8.09 (0.03)
	Range	7.50-7.84	7.50-7.79	8.07-8.10	8.06-8.11
	Median	7.81	7.77	8.07	8.10
pCO_2 (µatm)	Mean (± SD)	1122 (592)	1176 (500)	395(22)	384 (34)
	Range	752-1805	866-1753	370-413	360-423
	Median	810	909	402	370
HCO_3^- (µmol kg^{-1})	Mean (± SD)	2253 (98)	2246 (73)	1989 (23)	1968 (28)
	Range	2185-2365	2194-2330	1963-2006	1951-2001
	Median	2210	2214	2000	1953
CO_3^{2-} (µmol kg^{-1})	Mean (± SD)	111 (40)	104 (30)	215 (9)	225 (11)
	Range	66-139	70-125	209-227	212-232
	Median	129	117	211	231
Total Alkalinity (µmol kg^{-1})	Mean (± SD)	2527	2501	2520	2520

Table S2: ***Temperature and pCO_2 values recorded at the two nesting sites during the second experiment off Vulcano Island CO_2 seeps.***

		**Nesting sites**	
		**Ambient**	**High pCO_2**
T (°C)	Mean (± SD)	25.2 (0.3)	24.7 (0.3)
	Mean (± SD)	430 (50)	773 (213)
pCO_2 (µatm)	Range	333-530	443-1407
	Median	441	781

Table S3: ***Two-way PERMANOVA on the oxygen consumption rate (OCR) of embryos at different developmental stages (ST: initial, middle and late) collected from the two CO_2 nesting sites (Ambient and High). Significant differences are in bold.***

Source	df	SS	MS	Pseudo-F	p(perm)	Unique perms
Stage (ST)	2	255.24	127.62	99.773	**0.0001**	9952
CO_2	1	3.784	3.785	2.959	0.0903	9799
ST x CO_2	2	1.308	0.654	0.511	0.5976	9956
Residuals	63	80.584	1.279			
Total	68	339.67				

Table S4: ***PERMANOVA and PAIR-WISE t-tests on the oxygen consumption rate (OCR) of embryos at the late developmental stage in the six treatments. Significant differences are in bold.***

Source	df	SS	MS	Pseudo-F	p(perm)	Unique perms
Treatments	5	113.35	22.669	6.5573	**0.0001**	9937
Residuals	52	179.77	3.4571			
Total	57	293.12				

PAIR-WISE TESTS			
**Treatments**	**t**	**p(perm)**	**Unique perms**
High, Ambient	1.2403	0.236	5032
High, High-high	1.3166	0.2066	4994
High, Ambient-Ambient	1.6706	0.1232	5011
High, High-Ambient	0.3980	0.6957	9478
High, Ambient-High	2.3723	**0.0298**	9749
Ambient, High-high	0.0555	0.9533	4930
Ambient, Ambient-Ambient	0.1599	0.874	4994
Ambient, High-Ambient	1.3801	0.1851	9415
Ambient, Ambient-High	3.0805	**0.0057**	9773
High-high, Ambient-Ambient	0.1008	0.9158	5026
High-high, High-Ambient	1.4822	0.1533	9463
High-high, Ambient-High	3.1297	**0.0055**	9708
Ambient-Ambient, High-Ambient	2.0153	0.0602	8926
Ambient-Ambient, Ambient-High	3.119	**0.006**	9584
High-Ambient, Ambient-High	3.1651	**0.0052**	9827

Table S5: ***PERMANOVA and PAIR-WISE t-tests on the length at hatching of larvae after the six treatments. Significant differences are in bold.***						
**Source**	**df**	**SS**	**MS**	**Pseudo-F**	**p(perm)**	**Unique perms**
Treatments	5	0.6115	0.1223	3.3936	**0.0052**	9948
Residuals	408	14.704	0.0360			
Total	413	15.315				

**PAIR-WISE TESTS**			
**Treatments**	**t**	**p(perm)**	**Unique perms**
Ambient, High	0.3440	0.7251	658
High, High-high	0.3571	0.7211	679
High, Ambient-Ambient	1.3923	0.1709	761
High, High-Ambient	1.8802	0.0629	711
High, Ambient-High	1.2713	0.2026	646
Ambient, High-high	0.0842	0.9372	540
Ambient, Ambient-Ambient	1.0655	0.2823	608
Ambient, High-Ambient	1.6823	0.1003	563
Ambient, Ambient-High	2.0361	**0.0447**	483
High-high, Ambient-Ambient	1.4211	0.1582	669
High-high, High-Ambient	2.1615	**0.0348**	594
High-high, Ambient-High	2.3501	**0.0215**	543
Ambient-Ambient, High-Ambient	0.6437	0.525	659
Ambient-Ambient, Ambient-High	3.4512	**0.0005**	650
High-Ambient, Ambient-High	4.3323	**0.0001**	583

Table S6: ***PERMANOVA and PAIR-WISE t-tests on the yolk area of newly hatched larvae in the six treatments. Significant differences are in bold.***

Source	df	SS	MS	Pseudo-F	p(perm)	Unique perms
Treatments	5	0.0908	0.0182	34.485	**0.0001**	9952
Residuals	408	0.2149	0.0005			
Total	413	0.3057				

PAIR-WISE TESTS

Treatments	t	p(perm)	Unique perms
Ambient, High	2.9313	**0.0054**	248
High, High-high	8.6385	**0.0001**	5088
High, Ambient-Ambient	5.8236	**0.0001**	752
High, High-Ambient	11.241	**0.0001**	731
High, Ambient-High	10.673	**0.0001**	1007
Ambient, High-high	5.9511	**0.0001**	4656
Ambient, Ambient-Ambient	3.203	**0.0015**	668
Ambient, High-Ambient	8.0306	**0.0001**	625
Ambient, Ambient-High	7.9212	**0.0001**	871
High-high, Ambient-Ambient	3.2839	**0.0017**	5110
High-high, High-Ambient	0.0028	0.9976	4629
High-high, Ambient-High	2.3585	**0.0193**	5228
Ambient-Ambient, High-Ambient	3.6748	**0.0003**	765
Ambient-Ambient, Ambient-High	5.5909	**0.0001**	534
High-Ambient, Ambient-High	2.5551	**0.0123**	817
